# The Possibility of Applying the Vitamin D Brief Food Frequency Questionnaire as a Tool for a Country with No Vitamin D Data in Food Composition Tables

**DOI:** 10.3390/nu10091278

**Published:** 2018-09-10

**Authors:** Dominika Głąbska, Valentina Uroić, Dominika Guzek, Eva Pavić, Sandra Bival, Kamila Jaworska, Zlatko Giljević, Ewa Lange

**Affiliations:** 1 Department of Dietetics, Faculty of Human Nutrition and Consumer Sciences, Warsaw University of Life Sciences (SGGW-WULS), 159c Nowoursynowska Str., 02-787 Warsaw, Poland; kamila_jaworska@sggw.pl (K.J.); ewa_lange@sggw.pl (E.L.); 2 Department of Nutrition and Dietetics, University Hospital Centre Zagreb, 12 Kišpatićeva Str., 10-000 Zagreb, Croatia; valentina.uroic@kbc-zagreb.hr (V.U.); eva.pavic@kbc-zagreb.hr (E.P.); sandra.bival@kbc-zagreb.hr (S.B.); 3 Department of Organization and Consumption Economics, Faculty of Human Nutrition and Consumer Sciences, Warsaw University of Life Sciences (WULS-SGGW), 159c Nowoursynowska Str., 02-787 Warsaw, Poland; dominika_guzek@sggw.pl; 4 Division of Endocrinology, Department of Internal Medicine, University Hospital Centre, 12 Kišpatićeva Str., 10-000 Zagreb, Croatia; zlatko.giljevic@kbc-zagreb.hr; 5 Department of Internal Medicine, School of Medicine, University of Zagreb, 3 Šalata Str., 10-000 Zagreb, Croatia

**Keywords:** vitamin D, food frequency questionnaire, intake assessment, validation study, validity, reproducibility, VIDEO-FFQ

## Abstract

Although the role of vitamin D is well known, the possibility of assessing its intake may be constricted in countries with no vitamin D data in food composition tables, as in the case of Croatia. The aim of the presented study was to adjust the VIDEO-FFQ (Vitamin D Estimation Only—Food Frequency Questionnaire), previously validated in Poland, to the Croatian population and to assess the validity and reproducibility of the adjusted Cro-VIDEO-FFQ (Croatian—VIDEO-FFQ). The study involved a group of Croatian women aged 20–30 and the Polish questionnaire was adjusted for a population due to similarities of the nutritional habits between countries. 106 individuals were recruited and 63 completed all the stages of the study. Participants conducted a 3-day dietary record and filled out the Cro-VIDEO-FFQ1 (first stage), as well as the same questionnaire (Cro-VIDEO-FFQ2) 6 weeks after (second stage). The following vitamin D intakes were observed in the studied group: 1.9 µg (0.2–8.0 µg) for 3-day dietary record, 3.3 µg (1.1–10.6 µg) for Cro-VIDEO-FFQ1, 3.6 µg (1.4–7.8 µg) for Cro-VIDEO-FFQ2. The Bland-Altman indexes in assessment of validity and reproducibility were 4.8% and 6.3%, respectively, with mean differences of 0.55 µg and 0.12 µg, as well as limits of agreement −0.91–2.01 µg and −0.44–0.69 µg. The kappa coefficient indicated a fair agreement for validity (0.21) and substantial for reproducibility (0.62), while correlations were significant (*p* = 0.0027, *r* = 0.37 for validity; *p* < 0.0001, *r* = 0.80 for reproducibility). It was observed that VIDEO-FFQ may be adjusted as a simple tool to assess vitamin D intake in a population with no vitamin D data in food composition tables, while Cro-VIDEO-FFQ may be a valid tool for nutritional assessment in Croatia.

## 1. Introduction

The data from the ODIN Project (Food-Based Solutions for Optimal Vitamin D Nutrition and Health Through the Life Cycle Project) indicated that 13% of the European population is characterized by the serum 25-hydroxyvitamin D concentration lower than 30 nM/L, attributed to vitamin D deficiency [1]. However, the situation may differ depending on the country and its nutritional policy [2]. Vitamin D deficiency results from insufficient sun exposure, and as a result reduced vitamin D synthesis mediated by ultraviolet-B (UVB) radiation, as well as from low vitamin D dietary intake [3].

In general, the main source of vitamin D is the synthesis mediated by UVB radiation, but various sunlight exposure times and various angles of light rays because of the season and geographical position relative to the equator may cause insufficient synthesis [4]. At the same time, in European countries, sunshine-avoiding behaviors lead to the dietary intake becoming an even more significant source of vitamin D [5]. In the review by Schoor & Lips [5], it was reported that the serum 25-hydroxyvitamin D concentration is higher in Northern Europe (countries distant from the equator) than in Southern Europe (countries close to the equator), indicating the minor role of vitamin D synthesis for developed countries. Such a situation is caused by the dietary intake, which, in European countries, is mainly influenced by oil-rich fish intake and the intake of fortified products [6], contributing to the highest vitamin D dietary intake in Finland and Sweden, as reported by European Food Safety Authority (EFSA) [3] and confirmed by the recent reports [7,8].

However, for the Southern European countries, the fish intake is not limited by their geographical location, due to their general access to the coastline, but rather by the dietary habits, as the intake of fish products in Spain (42.4 kg/inhabitant per year) is comparable as the intake in Norway (53.4 kg/inhabitant per year), but in Greece, it is significantly lower (19.6 kg/inhabitant per year—data for 2011) [9]. Among such Southern European countries, which are characterized by low fish intake, there is Croatia, characterized by the intake of fish products of 19.7 kg/inhabitant per year [9]. For Croatia, it must be emphasized that the inadequate vitamin D dietary intake may be an especially serious problem, as the studies indicated the high prevalence of vitamin D deficiency in Croatian population [10], especially for women [11]. It is stated that despite the fact that the margarines are vitamin D fortified and some fortified dairy products are available, none of them contributes to the high vitamin D share in the diet. At the same time, there is no vitamin D data in the Croatian food composition tables [12], so the assessment of intake may be straightened.

Due to the lack of vitamin D data in the Croatian food composition tables, the typical assessment of intake conducted based on dietary record or recall, using the dietary software based on the food composition tables, must be for vitamin D replaced by other methods. The other method to assess the intake is the method of the food frequency questionnaire, which enables the assessment of the frequency of product consumption, and based on that, the assessment of nutrient intake as well [13]. Although there are some food frequency questionnaires developed to assess the vitamin D intake and validated for United States of America [14], Canada [15], United Arab Emirates [16], Saudi Arab [17], South Korea [18], Serbia [19], Poland [20], Finland [21], Sweden [22], United Kingdom [23], or Ireland [24], there is no such questionnaire developed and validated for Croatia.

The aim of the presented study was to adjust the VIDEO-FFQ (Vitamin D Estimation Only—Food Frequency Questionnaire), previously validated in Poland, to the Croatian population and to assess the validity and reproducibility of the adjusted Cro-VIDEO-FFQ questionnaire (Croatian—VIDEO-FFQ) in a group of Croatian women.

## 2. Materials and Methods

The study was approved by the Bioethical Commission of the National Food and Nutrition Institute in Warsaw (No. 0701/2015), and it was conducted in compliance with the guideline statements of the Declaration of Helsinki.

### 2.1. Adjusting the VIDEO-FFQ Questionnaire to the Croatian Population (Developing Cro-VIDEO-FFQ Questionnaire)

The food frequency questionnaire to assess the vitamin D intake in Croatian population was based on the VIDEO-FFQ questionnaire previously validated in Poland [20] and was designed in the international cooperation, while Croatian nutritionists participated in the adjustment for the Croatian population. The Polish questionnaire, included into the register of validated short dietary assessment methods by the National Cancer Institute/National Institutes of Health [25], was chosen as a basis for the study, due to several similarities in the Polish and Croatian diets, food products choice and the fact that both countries are the European Union members, causing similar legal regulations. The VIDEO-FFQ questionnaire was adapted to the typical Croatian dietary habits and products consumed in Croatia, based on the pilot study. The applied adjustments were as follows: (1) different serving sizes than in the original VIDEO-FFQ questionnaire, (2) dividing single questions into separate food items, (3) combining separate food items into a single question, (4) including an additional question about other fish consumed (other than listed in the original VIDEO-FFQ questionnaire), (5) including an additional question about vitamin D-enriched products consumed.

The final designed Cro-VIDEO-FFQ questionnaire included 22 food items with questions about the intake of food product groups, while only food products being the source of vitamin D were included, characterized by vitamin D content of at least 0.01 µg/100 g, as defined in the previous study [20]. To obtain better understanding of the structure of the questionnaire, food items were combined into seven food product groups, as in the case of the original VIDEO-FFQ questionnaire. The serving sizes, typical for the Croatian population, were applied, and in the Cro-VIDEO-FFQ questionnaire, they were expressed in grams and described using the typical household measures.

In the case of fish and fish products, respondents had to indicate, for each question, 1–2 most commonly consumed products from a group. Moreover, an additional question was included, as respondents were asked about the intake of other products from the group of fish that were not included in the questionnaire. It was especially important, as the food product list for fish was not adjusted for the Croatian population, and the fresh sardines, sprats, and catfish were indicated in a Croatian group (for 23.8% of respondents), which was not observed for Polish population (as sardines and sprats are consumed rather as processed ones in Poland, while a catfish is rarely consumed).

The frequency of intake was to be expressed in servings typically consumed in a day/ week/ month, during a previous year. Diversification of the period into day/week/month was applied, depending on the product, to facilitate the intake estimation for respondents. The respondents were also informed to specify the intake of products both used to prepare dishes and consumed between meals, as a snack, while indicating not only integers, but also decimal parts of servings being advised.

Vitamin D content in a serving specified in a questionnaire was during the analysis based on the calculation key elaborated for the VIDEO-FFQ questionnaire [20]. For the groups of fish and fish products, vitamin D intake was calculated based on the calculation key individually determined for each participant (as a mean value for the most commonly consumed products from each group), as in the case of the VIDEO-FFQ questionnaire [20]. In the case of the typical Croatian vitamin D-enriched products, it was based on the producer information on the packaging, while in the case of fish not listed in the original VIDEO-FFQ, the information about vitamin D content was based on the tables of nutritional value of food products elaborated by the Polish National Food and Nutrition Institute [26], as well as by the United States Department of Agriculture [27], if data were lacking. The whole procedure was verified during the pilot study.

Table 1 presents the food items included into a final Cro-VIDEO-FFQ questionnaire with serving sizes and frequencies, as well as the final vitamin D content in servings specified in the Cro-VIDEO-FFQ questionnaire. However, the final vitamin D content was not included into Cro-VIDEO-FFQ questionnaire, to not hamper the process of reliable data collecting.

### 2.2. Validation of the Cro-VIDEO-FFQ Questionnaire

The validation study was conducted in the period of eight months, since autumn to spring, to avoid answers being interfered by dietary habits modified during summer period, while the fish intake may be higher due to seaside holiday, higher availability of fresh ones and lower prices, as is also observed for other countries [28]. However, respondents were asked about the typical intake during the previous year to cover the whole year dietary habits. For each participant, the study participation took time of 6 weeks, as it was the necessary period between the first and second filling out of the questionnaire to assess its reproducibility.

The procedure of convenience sampling with the snowball effect was applied to conduct the validation, while young Caucasian women living in Zagreb, the capital of Croatia, were qualified, to enable personal interview. Young women were chosen as a future osteoporosis risk group, for which the vitamin D assessment is especially important [29]. Women were invited to participate via university social network, while they were informed about the inclusion criteria to participate, as well as about the exclusion criteria.

The inclusion criteria were as follows: (1) women, (2) aged 20–30 years, (3) living in Zagreb, (4) written consent agreement for participation in the study.

The exclusion criteria were as follows: (1) pregnancy, (2) lactation period, (3) following any special diet, (4) not completing the required forms of 3-day dietary record or Cro-VIDEO-FFQ questionnaire planned to be filled out twice.

**Figure 1:**
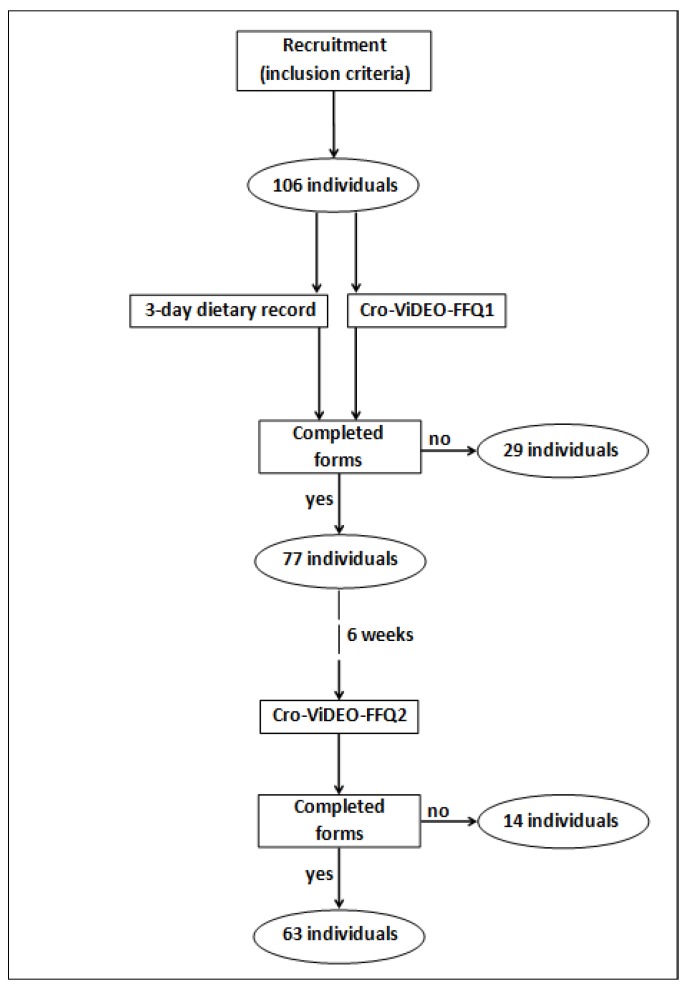
Study design and number of participants.

The number of respondents that were recruited and included to the first stage of the study was 106, but only 63 of them completed all the stages of the study (Figure 1). As the recommended sample size to conduct the validation of the food frequency questionnaire is at least 50–100 respondents [30], the obtained study group was interpreted as satisfactory.

The qualified respondents were asked to participate in two stages of the validation as follows: (1) the first stage: includes conducting a 3-day dietary record and filling out the Cro-VIDEO-FFQ questionnaire (indicated as Cro-VIDEO-FFQ1), in order to assess the validity of the questionnaire; (2) the second stage: includes filling out the Cro-VIDEO-FFQ questionnaire (indicated as Cro-VIDEO-FFQ2) for each respondent exactly 6 weeks after the first stage, in order to assess the reproducibility of the questionnaire.

The validity and reproducibility of the Cro-VIDEO-FFQ questionnaire were based on the definition by Willett and Lenart [31], and the validation process was conducted according to the same methodology as for the previously conducted validations [32,33]. As the aim of the study was to validate the food frequency questionnaire to analyze the diet against the other method enabling the analysis of diet, the biomarkers of vitamin D status were not included to assess the nutritional status, as well as the vitamin D supplementation that was applied by respondents was not included in the assessment. Taking it into account, the 3-day dietary record was chosen as a method of diet assessment as the self-reporting one, based on the recommendations of Cade et al. [30].

The 3-day dietary record was conducted by respondents during three typical, random, and not consecutive days, while two of them were to be week days, and one of them—a weekend day. Respondents received the structured form to note the meals with time and location of consumption and all the necessary information about consumed products—the dish ingredients and cooking technique, weight of serving (when packed products were consumed, or respondent had access to kitchen scale) or size of serving (estimated as standard household measures). Respondents were instructed how to conduct the dietary record, while they were informed of significance of scrupulous recording, reliable estimation, and not changing their typical dietary habits.

Basis on the obtained 3-day dietary records, the typical daily vitamin D intake was calculated, using the Energia 4.1. Polish dietary software with the information from the Polish tables of nutritional value of food products elaborated by the Polish National Food and Nutrition Institute [26] integrated. The Polish tables of nutritional value of food products were chosen to calculate the intake, as the VIDEO-FFQ questionnaire, being the basis of the developed Cro-VIDEO-FFQ questionnaire, was elaborated based on the indicated data, so to not interfere the conducted validation, the same data were used.

### 2.3. Statistical Analysis of Validation of the Cro-VIDEO-FFQ Questionnaire

During the analysis of validation, the following methods were applied independently, to assess both validity and reproducibility:

(1) analysis of the Bland-Altman plot: as the method for validation of the food frequency questionnaires recommended by Cade et al. [30], the mean difference, limit of agreement (LoA), as well as Bland-Altman index were calculated, after a log transformation applied due to non-parametric distribution, as commonly applied [34], while a Bland-Altman index of 5% was interpreted as a positive validation [35] and a Bland-Altman index of 10% was interpreted as a borderline significant [36], which was assumed a priori [37],

(2) analysis of cross-classification in quartiles: the share of correctly classified and grossly misclassified (classified to the opposite quartiles) individuals were indicated, while at least 50% of correctly classified and, at the same time, less than 10% of grossly misclassified individuals was interpreted as a positive validation [38];

(3) analysis of the weighted κ statistic: as a method recommended by Cade et al. [30] that may be applied to conduct the validation of the food frequency questionnaires involving a small number of ordered categories. The analysis was conducted on the basis of the quartile analysis and weighted κ statistic with linear weighting being applied, while the results were interpreted according to the criteria by Landis & Koch [39] in which the following values were indicated: lower than 0.20, a slight agreement; 0.21–0.40, a fair agreement; 0.41–0.60, a moderate agreement; 0.61–0.80, a substantial agreement; 0.81–1.0, an almost perfect agreement;

(4) analysis of the adequacy of intake: in comparison with the Estimated Average Requirement (EAR) level of 10 µg by Institute of Medicine [40], because there is no national recommendation of vitamin D intake in Croatia, while the share of correctly classified and misclassified (classified to the opposite categories) individuals were indicated;

(5) analysis of correlation: as the supplementary method that may be applied to conduct the validation of the food frequency questionnaires in conjunction with Bland-Altman method, recommended by Cade et al. [30], while Shapiro-Wilk test was applied to verify the normality of distribution as well as Spearman rank correlation coefficient (*r*) was analyzed, due to non-parametric distribution, and *r* higher than 0.5 was interpreted as a positive validation [38];

(6) analysis of the intraclass correlation coefficient (ICC): applied according to the method of Shrout & Fleiss [41], while the two-way mixed measures for the assessment of consistency was applied—ICC with 95% confidence interval (CI) was calculated and interpreted according to the criteria by Cicchetti [42], while values lower than 0.40, were indicated as a poor agreement; 0.40–0.59, a fair agreement; 0.60–0.74, a good agreement; 0.75–1.0, an excellent agreement.

Additionally, the Mann-Whitney U test was applied to compare the median values of vitamin D intake obtained from the 3-day dietary record and two food frequency questionnaires. The accepted level of significance was *p* ≤ 0.05. Using the following software, the statistical analysis was conducted: Statistica, version 8.0 (Statsoft Inc., Tulsa, OK, USA), Bland-Altman Statistica macro by Matt Coates, version 2009 (Statsoft Inc., Tulsa, OK, USA), PQStat, version 1.6.6. (PQStat Software SBO, Plewiska, Poland).

## 3. Results

Table 2 presents the intake of vitamin D observed for the analyzed group of Croatian women. The intake assessed using both the method of 3-day dietary record and the validated Cro-VIDEO-FFQ was presented, while the Cro-VIDEO-FFQ was applied twice. In comparison with the recommended level of 10 µg [40], it was observed that the dietary intake was inadequate for a vast majority of analyzed women. At the same time, the intake calculated based on the 3-day dietary record was lower than for Cro-VIDEO-FFQ1, while values observed for Cro-VIDEO-FFQ1 and Cro-VIDEO-FFQ2 did not differ.

Table 3 presents the contribution of product groups into the daily vitamin D intake observed for the analyzed group of Croatian women. The most important source of vitamin D was the group of fish and fish products (median of 44% of daily vitamin D intake), while less important were meat and meat products, eggs, and dairy products (10–17% of daily vitamin D intake), and cereal products as well as fats (including enriched ones) had a minor role.

The number of methods were applied during validation of applied Cro-VIDEO-FFQ (Table 4). For each applied method, the higher reproducibility than validity was observed. For the analysis of the Bland-Altman plot in the assessment of reproducibility, the observed LoA was narrower than in the case of the analysis of validity. At least 50% of correctly classified and, at the same time, less than 10% of grossly misclassified individuals were observed in the assessment of reproducibility for the analysis of the quartiles distribution, while the shares of 32% and 6%, respectively, were observed for the assessment of validity. A substantial agreement in the assessment of reproducibility and a fair agreement in the assessment of validity were stated for the analysis of the weighted κ statistics. For the analysis of the adequacy of intake, an excellent agreement was observed in the assessment of both reproducibility and validity, but in the assessment of reproducibility, all the assessed individuals were correctly classified. For the analysis of correlation, *r* higher than 0.5 was observed in the assessment of reproducibility, while it was lower in the assessment of validity. For the analysis of ICC, an excellent agreement in the assessment of reproducibility and a fair agreement in the assessment of validity were stated.

**Figure 2:**
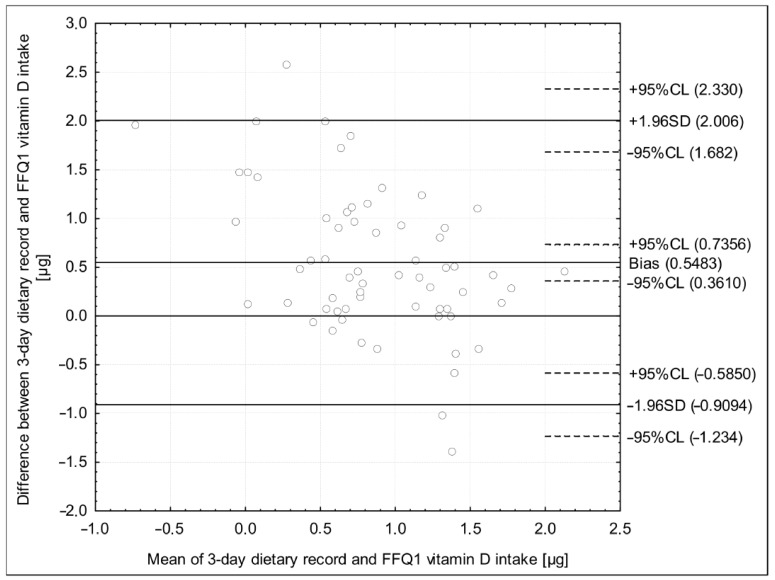
The Bland-Altman plot in the assessment of validity of the Cro-VIDEO-FFQ questionnaire (Bland-Altman index of 4.8%, due to 60 out of 63 individuals within the limit of agreement). Cro-VIDEO-FFQ1—the food frequency questionnaire to assess vitamin D intake in the first stage.

**Figure 3:**
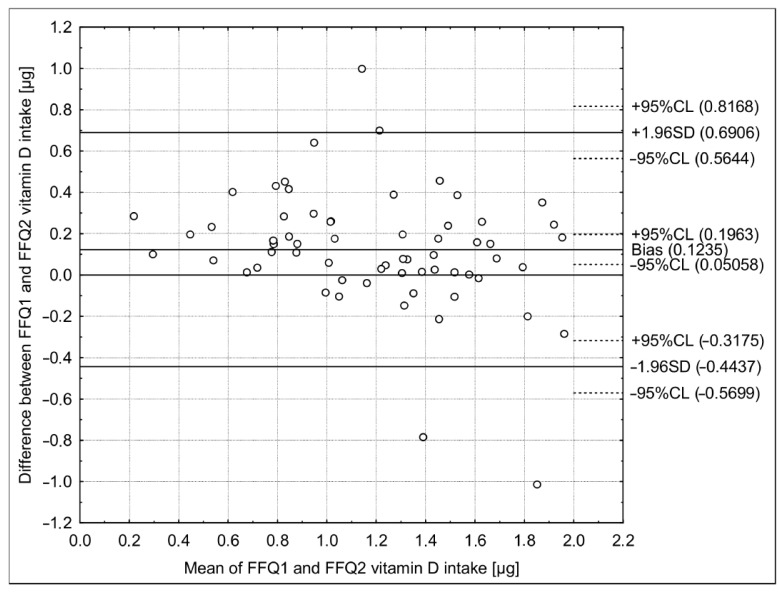
The Bland-Altman plot in the assessment of reproducibility of the Cro-VIDEO-FFQ questionnaire (Bland-Altman index of 6.3%, due to 59 out of 63 individuals within the limit of agreement). Cro-VIDEO-FFQ1—the food frequency questionnaire to assess vitamin D intake in the first stage Cro-VIDEO-FFQ2—the food frequency questionnaire to assess vitamin D intake in the second stage.

However, the further analysis of the Bland-Altman plots revealed that a Bland-Altman index lower than 5% was observed (Figure 2) in the assessment of validity, indicating a positive validation, while it was 6.3% in the assessment of reproducibility, which was also in agreement with the a priori assumption (Figure 3).

## 4. Discussion

The conducted statistical analysis revealed a satisfactory level of both validity and reproducibility of developed Cro-VIDEO-FFQ questionnaire and the fact that, in general, reproducibility was higher than validity for applied methods of assessment. However, it must be emphasized that a higher reproducibility than validity is commonly stated, and it was observed also for the original VIDEO-FFQ questionnaire validated in the Polish population [20].

Moreover, while the assessment of validity of the questionnaire is conducted, the accuracy of intake assessment for the reference method may be crucial. For vitamin D, the intake assessment is especially challenging, as vitamin D intake is characterized by a high day-to-day variation, due to the irregular fish intake [43], as the fish products are characterized by the highest content of vitamin D [27]. For such specific nutrients that are derived from the limited number of specific products, being rarely consumed (as fish products), the 24-h dietary recall is not a reliable method, and for a 3-day dietary record, there is also a risk that it will not capture a typical dietary pattern. As a result, the food frequency questionnaire may be a better method than dietary recall or dietary record to assess the vitamin D intake, as it covers a long period and may focus on specific products, being relevant for the vitamin D intake [22]. Similar observations are also indicated for other fish-derived nutrients [44]. Taking it into account, it must be emphasized, that the validity lower than reproducibility may have been observed not due to low accuracy in the case of food frequency questionnaire, but due to a low accuracy of vitamin D intake assessment in the case of dietary record. It also results from the fact, that validity is commonly lower than reproducibility, being observed in several validation studies [21,32,33,45,46].

Moreover, because vitamin D is derived from a limited number of products, the need to use the brief food frequency questionnaire, instead of the comprehensive one, is emphasized [22], as a higher number of food item questions in the comprehensive questionnaire may be associated with the higher error of intake estimation [47]. It may be confirmed by the fact, that the fish products were the main source of vitamin D for the assessed intake in the Croatian population, while less important were meat products, eggs and dairy products, and the others may be indicated as negligible.

In the case of the food frequency questionnaires, not only the questionnaire development and validation are significant, but also further adjustments for other populations and validations conducted in other populations are essential. Questionnaires, with their specific characteristics, should be developed for a specific country or region or even ethnic group [48], while the validation should be conducted in the target group [49].

The observed frequency of adequate dietary vitamin D intake was low—only one respondent was characterized by the adequate intake of it for the method of 3-day dietary record, while for food frequency questionnaires, it was observed for none of them. At the same time, the median of dietary intake was also very low, as it was 1.9 µg (0.2–8.0 µg) for 3-day dietary record, 3.3 µg (1.1–10.6 µg) for Cro-VIDEO-FFQ1 and 3.6 µg (1.4–7.8 µg) for Cro-VIDEO-FFQ2. The observed intake level may be compared with the level reported by EFSA for other countries [3] and it may be stated, that the level observed for young women in Croatia is comparable with the level stated for young women in Germany (2.0–2.6 µg) [50], Austria (2.1–3.3 µg) [51], Ireland (2.4 µg) [52], Netherlands (2.6 µg) [53] and Denmark (2.6–2.8 µg) [54].

The analysis of vitamin D sources in the analyzed Croatian population may be conducted. Moreover, based on the main vitamin D sources, the role of the specific questionnaire for each population may be justified. It was observed in our study for Croatian population, and different sources of the same nutrient observed in other populations, due to various dietary habits and accessibility to various products [55]. In our study, the main sources of vitamin D for young women were fish products, meat products, eggs, and dairy products. At the same time, while other questionnaires were used, Bärebring et al. [22] observed fish products, spread margarine, and dairy products (including fortified ones) mainly as vitamin D sources for Swedish young women population, while Zareef et al. [17] observed dairy products (including fortified ones) and fish products as the less important for Saudi Arabian young women. The main difference in comparison with other populations is the role of meat products as a source of vitamin D in the Croatian population, although the general vitamin D content in meat products is rather low [27]. However, a similar situation was observed in the United States of America and Canada [56]. While fish product intake is low, the role of other products characterized by lower vitamin D content, but a high intake, is becoming prominent. In countries characterized by a high fish intake, such as Japan, they are the main source of vitamin D, and other products may be treated as minor [57].

Hence, the possibilities to apply the questionnaire to assess the intake of specific nutrient in a specific group should not be extrapolated to the other nutrients and other population groups. It was the reason the VIDEO-FFQ questionnaire was adjusted for Croatian population and Cro-VIDEO-FFQ questionnaire was validated in a Croatian population. In the conducted study, including not only fish products and dairy products, including fortified ones (commonly indicated as a sources of vitamin D) to the Cro-VIDEO-FFQ questionnaire, but also other products, including meat products, allowed to capture the main sources of assessed nutrient. However, because fish products, if consumed, contribute to the high intake of vitamin D, they may be the main source of errors while the intake is assessed based on the dietary recall or record [45].

Especially for vitamin D, the assessment of intake is very important not only due to commonly observed too low intake [3], but also because even fortification does not guarantee the intake correction and so the intake must be monitored [17]. To conduct such monitoring of intake, the food frequency questionnaires may be applied if they are validated and the results of validation are satisfactory. The observed results of validation of Cro-VIDEO-FFQ questionnaire may be indicated as satisfactory, or very good, in the case of assessment of reproducibility. Only in the case of analysis of the Bland-Altman plot, the results of validity were slightly better than in the case of reproducibility, but it must be interpreted as resulting from the specificity of the method. As for validity, 60 individuals out of 63 were within the LoA, and for reproducibility, 59 of them were within the LoA, only 1 individual contributed to the difference of observed Bland-Altman index, which, in both cases, was lower than 10% (as assumed a priori as necessary for positive validation [37]); but for validity, it was even lower than 5%.

The validation was conducted for a group of women, but the further validation for men is needed; however, in most studies it is stated that for developed countries the vitamin D intake is not associated with the gender [6]. Moreover, for the further study the sociodemographic status should be included to analyze the association with the income level, due to general high price of fish products.

Such a positive validation, as in the presented study, indicates that the validated Cro-VIDEO-FFQ questionnaire may be applied to assess the vitamin D intake in the Croatian young women population, which was previously indicated as necessary due to general high prevalence of vitamin D deficiency in a Croatian population [10] and due to the need to monitor the intake [17]. Moreover, the lack of vitamin D data in the Croatian food composition tables imposes such actions for the public health purposes. However, the conducted study also indicates the prospective possibility for other countries to include the questionnaire assessment of vitamin D intake, which may be even more reliable than the assessment conducted based on dietary recall or record, due to specific sources of vitamin D.

## 5. Conclusions

The Cro-VIDEO-FFQ questionnaire developed to assess the vitamin D intake in the Croatian population was characterized by a satisfactory validity and reproducibility level for women, while the reproducibility was even higher than validity.

The observed vitamin D sources in a Croatian population indicate that in countries characterized by a low fish intake, the questionnaire to assess the vitamin D intake should include the products other than fish that may contribute to a significant share of the nutrient.

The Cro-VIDEO-FFQ questionnaire may be a valid tool to assess the vitamin D intake in the Croatian population and should be applied, due to the lack of vitamin D data in the Croatian food composition tables.

## Figures and Tables

**Table 1:** The food items included into Cro-VIDEO-FFQ questionnaire to assess the vitamin D intake in a Croatian population accompanied by the vitamin D content in food items included.

The Food Items Included into Cro-VIDEO-FFQ Questionnaire				The Vitamin D Content Per 1 Serving (µg)	
Group of Products	Products	Serving Size	Frequency		
Fresh and smoked fish	Salmon, rainbow trout, herring, eel	50 g (deck of cards)	Monthly	Salmon	7.50
				Rainbow trout	7.80
				Herring	9.50
				Eel	15.00
	Halibut, mackerel, brook trout, sole, tuna	50 g (deck of cards)	Monthly	Halibut	2.50
				Mackerel	2.50
				Brook trout	1.05
				Sole	4.00
				Tuna	3.60
	Cod, flounder, plaice, pollock, hake, bass, zander, pike	50 g (deck of cards)	Monthly	Cod	0.50
				Flounder	0.40
				Plaice	0.40
				Pollock	0.50
				Hake	0.50
				Bass	0.40
				Zander	0.35
				Pike	0.45
	Other fish (to be specified)	50 g (deck of cards)	Monthly	Depending on the product	
Fish products	Herrings, sardines, and tuna products	100 g (e.g., 2 rollmops, small can of tuna, 2/3 of can of herrings)	Monthly	12.36	
	Other fish products	100 g (e.g., 1/3 of can of fish stew)		0.93	
Dairy products	Milk and milk beverages (yoghurt, kefir, buttermilk, cream)	250 g (1 glass)	Weekly	0.28	
	Vitamin D fortified products (to be specified)	250 g (1 glass)		Depending on the product	
	Rennet, blue and soft penicillium cheese	20 g (1 slice)		0.09	
	Feta cheese	15 g (1 slice)		0.08	
	Cottage cheese	50 g (1 thick slice, 2 tablespoons)		0.08	
	Processed cheese	25 g (1 slice, 1 spoon, 1 triangle serving)		0.07	
	Homogenized cheese	150 g (1 package)		0.23	
	Dairy ice cream	40 g (1 scoop)	Monthly	0.30	
Eggs	Egg	50 g (1 medium egg)	Weekly	0.85	
	Egg yolk	20 g (1 yolk)		0.90	
Meat and meat products	Meat	100 g (palm of small hand)	Weekly	0.75	
	Meat products	15 g (thin slice of ham, 3 slices of sausage)		0.09	
Cereals	White wheat and confectionery bread	35 g (1 slice, small roll)	Weekly	0.06	
	Cooked egg pasta	100 g of cooked (1 glass)		0.25	
Fats	Butter, butter products, pork fat	5 g (1 teaspoon)	Daily	0.03	
	Fortified margarine	5 g (1 teaspoon)		0.31	

Cro-VIDEO-FFQ: Croatian—Vitamin D Estimation Only—Food Frequency Questionnaire.

**Table 2:** The observed vitamin D intake and the assessment of its adequacy.

		3-Day Dietary Record	Cro-VIDEO-FFQ1 *	Cro-VIDEO-FFQ2 *
Mean ± standard deviation (µg)		2.4 ± 1.7	3.5 ± 1.8	3.8 ± 1.5
Median (µg)		1.9 **	3.3 **	3.6 **
Minimum (µg)		0.2	1.1	1.4
Maximum ([µg)		8.0	10.6	7.8
Individuals characterized by adequate intake in comparison with EAR level [40]	N	1	0	0
	[%]	1.6	0	0
Individuals characterized by inadequate intake in comparison with EAR level [40]	N	62	63	63
	[%]	98.4	100	100

* for comparison between 3-day dietary record and Cro-VIDEO-FFQ1: *p* = 0.0000 (Mann-Whitney U test) and for comparison between Cro-VIDEO-FFQ1 and Cro-VIDEO-FFQ2: *p* = 0.1292 (Mann-Whitney U test); ** distribution different than normal (Shapiro-Wilk test—*p* ≤ 0.05) Cro-VIDEO-FFQ1—the food frequency questionnaire to assess vitamin D intake in the first stage Cro-VIDEO-FFQ2—the food frequency questionnaire to assess vitamin D intake in the second stage.

**Table 3:** The contribution of product groups into the daily vitamin D intake calculated based on the Cro-VIDEO-FFQ1 questionnaire (the food frequency questionnaire to assess vitamin D intake in the first stage).

Group of Products	Share of Vitamin D Intake (%)			Vitamin D Intake (µg)		
	Mean ± Standard Deviation	Median	Minimum–Maximum	Mean ± Standard Deviation	Median	Minimum–Maximum
Fish and fish products	43.2 ± 26.0	43.8 *	0–96.6	1.8 ± 1.9	1.4 *	0–9.9
Meat and meat products	20.4 ± 13.4	17.2 *	0–59.6	0.6 ± 0.3	0.6 *	0–1.3
Eggs	14.3 ± 12.1	10.7 *	0–68.8	0.5 ± 0.8	0.4 *	0–6.6
Dairy products	10.8 ± 7.2	10.0 *	0–37.2	0.3 ± 0.2	0.3 *	0–0.9
Cereal products	4.2 ± 5.0	3.2 *	0–39.3	0.1 ± 0.3	0.1 *	0–2.3
Fats	7.1 ± 9.8	2.4 *	0–38.8	0.2 ± 0.4	0.1 *	0–1.9

* distribution different than normal (Shapiro-Wilk test—*p* ≤ 0.05).

**Table 4:** The validation of the Cro-VIDEO-FFQ questionnaire including assessment of validity and reproducibility.

The Assessed Parameters		Analysis of Validity—Cro-VIDEO-FFQ1 vs. 3-Day Dietary Record	Analysis of Reproducibility—Cro-VIDEO-FFQ1 vs. Cro-VIDEO-FFQ2
Bland-Altman plot analysis	Mean difference	0.55	0.12
	Limit of agreement (LoA)	−0.91–2.01	−0.44–0.69
Analysis of quartiles	Individuals correctly classified	20 (32%)	47 (75%)
	Individuals grossly misclassified	4 (6%)	1 (2%)
	Weighted κ statistic	0.21	0.62
Analysis of adequacy in comparison with EAR level [40]	Individuals correctly classified	62 (98.4%)	63 (100%)
	Individuals misclassified	1 (1.6%)	0 (0%)
Analysis of correlation	*p*-Value	0.0027	<0.0001
	*r* Spearman correlation coefficient	0.37	0.80
Intraclass correlation coefficient (ICC)	ICC	0.56	0.81
	95% confidence interval (CI)	0.27–0.73	0.69–0.89

Cro-VIDEO-FFQ1—the food frequency questionnaire to assess vitamin D intake in the first stage; Cro-VIDEO-FFQ2—the food frequency questionnaire to assess vitamin D intake in the second stage.
